# Co-design of the Implementation Toolkit for Small and Sick Newborn Care: a global open-access knowledge management web platform and linked community targeting the know–do gap

**DOI:** 10.1186/s12887-026-07420-2

**Published:** 2026-08-24

**Authors:** Lauren E. Allison, Mbozu Sipalo, Tobias Whatley, Zoe Griffiths, David Gathara, Sarah Murless-Collins, Chinyere Ezeaka, Olufunke Bolaji, Msandeni Chiume, Nahya Salim, Karen Walker, Alex Stevenson, Ralph Hale, Ousmane Ndiaye, Hema Magge, Massimo Salvadori, Federica Cassera, Neena Khadka, Tedbabe Degefie Hailegebriel, Rebecca Richards-Kortum, Maria Oden, Ornella Lincetto, Sara Liaghati-Mobarhan, Harriet Ruysen, Olive Cocoman, Anita Gibson, Gagan Gupta, Joy E. Lawn, the Implementation Toolkit for Small and Sick Newborn Care Co-Design Group, Aeesha N. J. Malik, Eric O. Ohuma, Geoffrey Manda, Ifeanyichukwu Anthony Ogueji, Irabi Kassim, John Wainaina, Josephat Mutakyamilwa, Josephine Shabani, Karim Manji, Louise Tina Day, Nahya Salim Masoud, Olabisi Dosunmu, Rebecca E. Penzias, Samuel Ngwala, Carole Kenner, Donat Shamba, Evelyn Zimba, Fatima Gohar, Gaurav Sharma, Mary Kinney, Meghan Kumar, Richard Kagimu, Timothy Powell-Jackson, Anniina Lockwood, Charles Osuagwu, Edith Gicheha, Ekran Rashid, Ephraim Kumi Senkyire, George Banda, Goldy Mazia, Grace Tahuna Soko, Jennifer Werdenberg-Hall, Merran Thomson, Ruth Davidge, Elizabeth Ngowi, Hamish R. Graham, James H. Cross, Joseph Bailey, June Madete, Megan Heenan, Patricia Coffey, Robert Tillya, Ryan Johnston, Taylor Boles, Alison Morgan, Prarthna Dayal, Opeyemi Odedere, Chris Harnish, Christine A. Bohne, Georgina Msemo, Harish Chellani, Kondwani Kawaza, Padraic Casserly, Prateek Gupta, Jaya Chandna, Kiersten Israel-Ballard, Maureen Majamanda, Rosie Steege, Silke Mader, Elizabeth Molyneux, Naomi E. Spotswood, Caroline Kinsella, Laura Fitzgerald, Robyn Churchill, Shanon McNab, Suzanne Stalls, Aduragbemi Banke-Thomas, Loveday Penn-Kekana, Peter Acker, Cally Tann, Samantha Sadoo, Kalpana Upadhyay Subedi, Clare Gilbert, Natalie Dottle Mitchell

**Affiliations:** 1 Department of Infectious Disease Epidemiology and International Health, London School of Hygiene & Tropical Medicine, Keppel Street, London, WC1E 7HT UK; 2 Girls Not Brides, The Global Partnership to End Child Marriage, 27 Old Gloucester Street, London, WC1N 3AX UK; 3 KEMRI-Wellcome Trust Research Programme, Hospital Road, Kilifi, Kenya; 4 Department of Paediatrics, College of Medicine, University of Lagos, Lagos, Nigeria; 5 Afe Babalola University, Km 8.5 Afe Babalola Way, Ado-Ekiti, Ekiti State Nigeria; 6 Paediatrics and Child Health Department, Kamuzu Central Hospital, Lilongwe, Central Region 207233 Malawi; 7 University College London, Gower Street, London, WC1E 6BT UK; 8 Muhimbili University of Health and Allied Sciences, United Nations Rd, Dar Es Salaam, Tanzania; 9 Council of International Neonatal Nurses, Yardley, PA USA; 10 African Neonatal Association, KG 620 Street ST9, Kigali, Rwanda; 11 Cheikh Anta Diop University, Dakar-Fann, Dakar, BP 5005 Senegal; 12 Gates Foundation, 500 5Th Ave N, Seattle, WA 98109 USA; 13 Paolo Chiesi Foundation, Via Paradigna 131/A, Parma, 43122 Italy; 14 Independent Researcher, 4515 Willard Avenue, Chevy Chase, MD 20815 USA; 15 UNICEF, Sudan Country Office, Gereif West, Block 3, Hara 1/D, Omak Street, East Khartoum, Manshiya, Sudan; 16 Rice University, 6100 Main St, Houston, TX 77005 USA; 17 Independent Researcher, Via Della Vitalba 8, Trieste, 34151 Italy; 18 United Nations Children’s Fund, Malawi, Blantyre, 52VQ+VCP Malawi; 19 Laerdal Global Health, Stavanger, Norway; 20 AlignMNH/Jhpiego, Washington, DC 20036 USA; 21 UNICEF Kenya Country Office, P.O. Box, Nairobi, 44145 - 00100 Kenya

**Keywords:** Newborn, Neonatal, Low- and Middle-Income Countries, Inpatient Care, Knowledge Management, Digital Health, Evidence-based Implementation

## Abstract

**Background:**

Most births worldwide (> 80%) occur in health care facilities, yet 2.3 million newborns die annually. If the *know*–*do* gap between evidence and implementation was closed, an estimated 752,000 newborn deaths could be prevented per year. We describe the co-design of the Implementation Toolkit (Newborn Toolkit) for Small and Sick Newborn Care (SSNC), a web platform and linked community of global implementers, facilitated by NEST360 and UNICEF to enable access to curated resources, including guidelines, practical tools and peer learning for contextual adaptation.

**Methods:**

A systematic three step co-design process was followed. Step 1) Structure: We used an organising framework of WHO and UNICEF ten core components for systems strengthening for SSNC. We then reviewed relevant knowledge management platforms to identify elements facilitating user engagement. Step 2) Content: > 300 implementers collated publications and tools according to the ten core components. Step 3) Refining and building community: User data analytics plus direct feedback from the global communities of practice provided data to improve website content and community uptake, notably regular webinars.

**Results:**

Step 1) Structure: In 2020, the Newborn Toolkit website structure was co-designed based on the ten core components. Step 2) Content: Working groups, organised by core components, collated over 1,100 resources in 15 languages. Step 3) Refining and building community: Cross-country learning was facilitated through 45 webinars with multi-disciplinary speakers from all continents including caregivers, clinicians, non-governmental organisation representatives, engineers, and data scientists. French language translation and engagement was added between 2023–2025. Unique user counts increased with 28,146 in 2023 to 62,561 in 2025 from 198 countries and territories. The most viewed content includes WHO guidelines, neonatal floor plans, the ABC device costing tool, and data tools.

**Conclusions:**

Given the urgency for accelerated progress for newborn survival by 2030, faster implementation of proven solutions is needed. It is crucial that implementers can access evidence and tools to adapt for their specific context, rather than “reinventing the wheel”. Systems change is complex, requiring novel approaches to make it doable, such as standard, simplified action pathways available on the Newborn Toolkit. Gaps to address include evidence availability in multiple languages.

## Key findings

### What was known?

- 2.3 million neonatal deaths are estimated annually and 65 countries are at-risk of missing Sustainable Development Goal 3.2 of reducing the number of neonatal deaths to less than 12 per 1,000 live births by 2030
- There exists a *know–do* gap in global newborn health referring to the inability to translate evidence, what is known to work, into practice to improve health outcomes for WHO-UNICEF level-2 Small and Sick Newborn Care
- Frameworks based on country experiences have been created to guide health systems strengthening such as the WHO/UNICEF core components; however, operationalisation is needed
- Evidence, guidelines, and resources for Small and Sick Newborn Care were available; however, there existed a gap in knowledge management platforms dedicated to enabling evidence-based implementation of health systems strengthening interventions in low-resource settings

### What was done that is new?

- We co-designed a knowledge management platform for resources including tools, readings, and case studies to enable evidence sharing for Small and Sick Newborn Care in low-resource settings
- Content and resources on the site were organised according to the ten WHO/UNICEF core components for health systems strengthening plus infection prevention and control
- This platform was operationalised and scaled by linking to, and engaging with, a global community of practice
- We applied a continuous learning and feedback integration approach to develop and refine content, enable accessibility including by language, and tailor engagement initiatives to implementer needs informed by website user and webinar data analytics

### What was found?

- The platform hosts over 1,100 tools and readings with resources available in 15 languages
- Between 2021–2025, this platform had 157,452 unique users, from 198 countries and territories
- 45 webinars were hosted between 2022–2025 showcasing implementation case studies and facilitating cross-country learning
- Knowledge management and targeted engagement can enable uptake of information by organising content in practical, digestible, and feasible formats

### What next?

- The *know–do* gap in Small and Sick Newborn Care needs to be closed. Providing up-to-date, relevant evidence that adapts to emerging knowledge and local learning, alongside leadership and financial inputs, could facilitate quality care delivery through data-driven decision making
- Bridging language barriers, through content translation, is a priority and crucial to enable wider access to up-to-date evidence 

## Background

Two million three hundred thousand newborn deaths occur annually with sub-Saharan Africa and South Asia accounting for the highest burden. Prematurity is the leading cause of death globally among neonates followed by birth asphyxia/trauma, congenital abnormalities, lower respiratory infections, and sepsis [1]. With appropriate intervention 75% of these deaths can be prevented [2].

The majority of births globally take place in health care facilities; 80% of births in sub-Saharan Africa take place in facilities [3]. Yet many facilities lack adequate capacity to deliver Essential Newborn Care (ENC), defined as the critical care for all babies in the first days after birth including immediate care at birth, thermal care, resuscitation, breastfeeding, infection prevention, response and recognition to danger signs, and referral [4]. Similarly, facility capacity is also lacking for level-2 Small and Sick Newborn Care (SSNC) interventions, defined as specialised care for newborns who are born preterm, underweight, or develop medical and surgical complications, such as assisted feeding, oxygen, and detection and management of neonatal conditions [5]. To reduce preventable deaths through delivery of ENC and SSNC within high burden settings a health systems strengthening approach is required.

The World Health Organization (WHO) and United Nations Children’s Fund (UNICEF), based on learnings from countries that have systemically scaled up SSNC units in the last decade, have developed a framework to guide other countries in the scale up of SSNC with ten core components for health systems strengthening: leadership and governance, financing, human resources, infrastructure, equipment and commodities, data systems, functional referral systems, linkages to maternal care, family and community, and post discharge follow-up [6]. However, criticisms of this framework include a lack of representation of the dynamism and interaction between core components [7, 8]. Notably, practical implementation of health systems interventions draw on various core components with strategies for implementation needing to consider a holistic systems approach [9]. For instance, practical implementation of specific interventions, such as respiratory support, requires integration of human resources training, financing, and information systems to monitor change. To make health systems strengthening actionable, operationalising this framework is needed, with consideration to the dynamism of health systems components to guide implementation of evidence and quality standards into practice.

A robust evidence base exists to inform essential SSNC interventions; however, strategies are needed to enable uptake of evidence in practice for health systems change which is often complex and requires localisation for the diverse settings in which SSNC is provided [10, 11]. In global health, the *know*–*do* gap*,* also called the *theory–practice* gap, refers to the inability to translate evidence, what is known to work, into real world practice [12, 13]. This lack of evidence-based implementation affects quality of care and ultimately, outcomes. Poor quality care is a bigger burden to mortality reduction than lack of access, with 60% of deaths from conditions amenable to health care due to poor-quality care [10]. Poor dissemination of research findings has been reported as an impediment to evidence implementation by policy makers [14, 15].

Knowledge management is a means to provide the right information, to the right person, at the right time [16]. Knowledge management encapsulates knowledge generation, storage, processing, transfer, and utilisation [17]. The number of knowledge management platforms for health have increased in recent years with a range of focuses including evidence networks, surveillance tools, observatories, and data platforms [18, 19]. However, there were no knowledge management platforms dedicated to enabling evidence dissemination for SSNC in low-resource settings. We aimed to enable knowledge sharing through the creation of an online open-access resource platform and linked community — The Implementation Toolkit for Small and Sick Newborn Care, also known as the Newborn Toolkit.

### Aim and objectives

This paper is part of a supplement from Newborn Essential Solutions and Technologies 360 (NEST360), an alliance of partners, including five African governments (Kenya, Malawi, Nigeria, and Tanzania, Ethiopia), working to reduce neonatal inpatient deaths by improving newborn care in hospitals through device installation, training, and quality improvement. In this paper we aim to describe the systematic process of co-designing and refining an open-access online platform for knowledge sharing in SSNC.

Our objectives were:

- Objective 1: Review the landscape of existing knowledge management platforms focused on health systems change. Identify and describe key elements and structure that facilitate knowledge organisation and user-friendly engagement with evidence.
- Objective 2: Co-design platform development and curation of existing evidence for SSNC, organised according to WHO and UNICEF’s core components for health systems strengthening, plus infection prevention and control.
- Objective 3: Refine content and operationalise engagement with a global community of practice to enable evidence sharing.

## Methods

The Implementation Toolkit for SSNC, an open access online resource platform, was developed in collaboration with UNICEF as part of the NEST360 multi-country alliance for neonatal mortality reduction. We adopted a systematic approach comprising three steps: 1) Reviewing existing open access platforms and stakeholder consultations, 2) co-designed platform development, content organisation, refinement, and translation, and 3) deploying engagement initiatives across social networks targeting a global community of SSNC implementers.

### Methods by objectives

#### Objective 1

Platform structure and development was informed by 1) searching for and reviewing existing open access knowledge management platforms relevant to global health and neonatal health topics and 2) consulting a multidisciplinary group of newborn care implementers in low- and middle- income countries LMIC. We partnered with a website development company to design and refine the layout of the web platform based on *a priori* criteria for platform structure and content organisation.

##### Focus on implementation

Content on the site needed to facilitate implementation of evidence into practice for level-2 SSNC in low-resource settings.

##### Multi-level systems approach

A multi-level systems approach was taken to account for inputs to health care delivery beyond the facility level such as national leadership, governance, and financial mechanisms impacting on health delivery. The platform needed to host evidence across all elements of system change in one accessible location.

##### Neutral and consistent branding

The platform was branded with a colour palette unique to the Newborn Toolkit and a neutral website domain (www.newborntoolkit.org) with the intention of signalling a collaborative ownership with the global community of implementers and democratising access to evidence.

##### Open-access and accessibility

All learning including content, tools, publications, and webinars needed to be open-access to reduce knowledge sharing barriers. As the target audience for this platform includes those based in low-resource settings, this platform needed to be accessible on mobile and desktop devices and use low bandwidth.

##### Ownership and representation

The platform needed to appeal to a wide range of implementers including importantly parent groups as well as policy makers, physicians, nurses, midwives, researchers, funders, biomedical engineers, among others. Mechanisms for community members to give feedback on platform design was a key requirement.

#### Objective 2

Development of the platform and review of potential content occurred in a staged process: 1) content curation and organisation by core component working groups, 2) editorial team revision, 3) Search Engine Optimisation (SEO) and validation, and 4) French language translation.

To achieve this, eleven working groups were established (one for each core component), with working group members identified for their related practical experience of implementation and research in this field.

Working group members identified, screened, triaged, and organised content, including reports, checklists, floor plans, strategies, investment cases, within their Toolkit section alongside developing a narrative overview of the topic. Content was assessed for inclusion with consideration to quality (peer reviewed, reputable sources), practical guidance, focused on high burden settings for neonatal morbidity and mortality, and in alignment with WHO and United Nations recommendations.

An editorial team comprised of representatives from NEST360 (JEL, ZG, and DG) and UNICEF (GG), ensured messaging and flow were cohesive across content curated by different working groups. A website content coordinator (TW) reviewed and refined all content to ensure it complied with Search Engine Optimisation (SEO) and best web accessibility practices. Content on the site was translated to French using the artificial intelligence-enabled Word Press Multi-lingual (WPML) Plugin. Post-translation review was conducted by a French-language speaker and health care professional to ensure accuracy and meaning were preserved.

#### Objective 3

Engagement with key constituents is necessary for knowledge translation and was central to the Implementation Toolkit for SSNC strategy. Engagement efforts were tailored to reach high-burden settings for neonatal mortality including sub-Saharan Africa and South Asia.

#### Engagement initiatives

Between November 2021 and December 2025, we implemented the following outreach engagement strategies:

A monthly webinar series was created featuring multi-country and multi-lingual learning. Each webinar theme was aligned to one of the ten core components. Live translations of webinars were provided from English to French. Webinars followed a systematic format including a topic overview and usually three examples of local, national, or regional learning across different regions worldwide. Webinars focused on sharing real world examples of implementation. A question-and-answer period was moderated to enable multi-directional learning opportunities.

Social media profiles and campaigns were developed for the Toolkit. LinkedIn, Facebook, X (formerly Twitter) and Bluesky were selected as main engagement platforms with a higher presence of relevant professional and caregiver representation.

A monthly newsletter was developed using Mailchimp to share key updates on newborn health and Toolkit-related initiatives. These include announcements of upcoming and recent webinars, new guidance or research, relevant news items, and blogs.

An expert advisory team was created with members representing clinical care, policy makers, funders, researchers, professional associations, and caregivers. Between 2022–2025, this group met quarterly to strengthen inter-disciplinary and cross-sectoral collaboration, review engagement activities, and inform the strategic direction of the Toolkit and linked community.

An annual crowdsourcing essay contest was developed by the expert advisory team in 2023 to commemorate World Prematurity Day and promote global discussion on key SSNC themes. Essay topics have focused on making the case for investing in SSNC, advancing family-centred care, and cross-professional neonatal collaboration to improve quality of care.

#### Data analytics

Monthly data analytics reports were developed with indicators to track user engagement and site growth. Website analytics included Google search engine impressions, average search engine position, 28-day active users, new users, route of accessing the site, type of device used to access the site, geographical distribution of users, page views, and length of engagement. Active users are defined according to Google Analytics as a unique website visitor who had an engaged session on the site. Criteria for an engaged session includes one of the following: a user session on the website that lasts longer than 10 s, triggers a ‘key event’ such as interacting with a site resource, or users that generate greater than two page views [20]. Google Analytics automatically excludes known bot traffic [21]. Additional site security measures such as Fail2ban an intrusion prevention software framework as well as server-level firewall rules restricting access to only authorised ports and services were implemented to further limit bot engagement. Monthly webinar attendance and social media growth and engagement was also monitored.

## Results

### Results by objectives

#### Objective 1

A multi-level knowledge management structure was taken to organise content and resources. The platform was organised using the WHO/UNICEF ten core components for health systems strengthening (plus infection prevention and control) providing a specific landing page for each of the of the core components.

The main body of each core component contains narrative style topic overview and sub-sections with links to related publications and tools enabling easy access to resources. Tools and readings can be filtered according to language, publication year, and topic. Topics are organised into UN Guidelines, Single-Country Evidence, and Multi-Country Evidence.

#### Design and Toolkit Cog

**Fig. 1:**
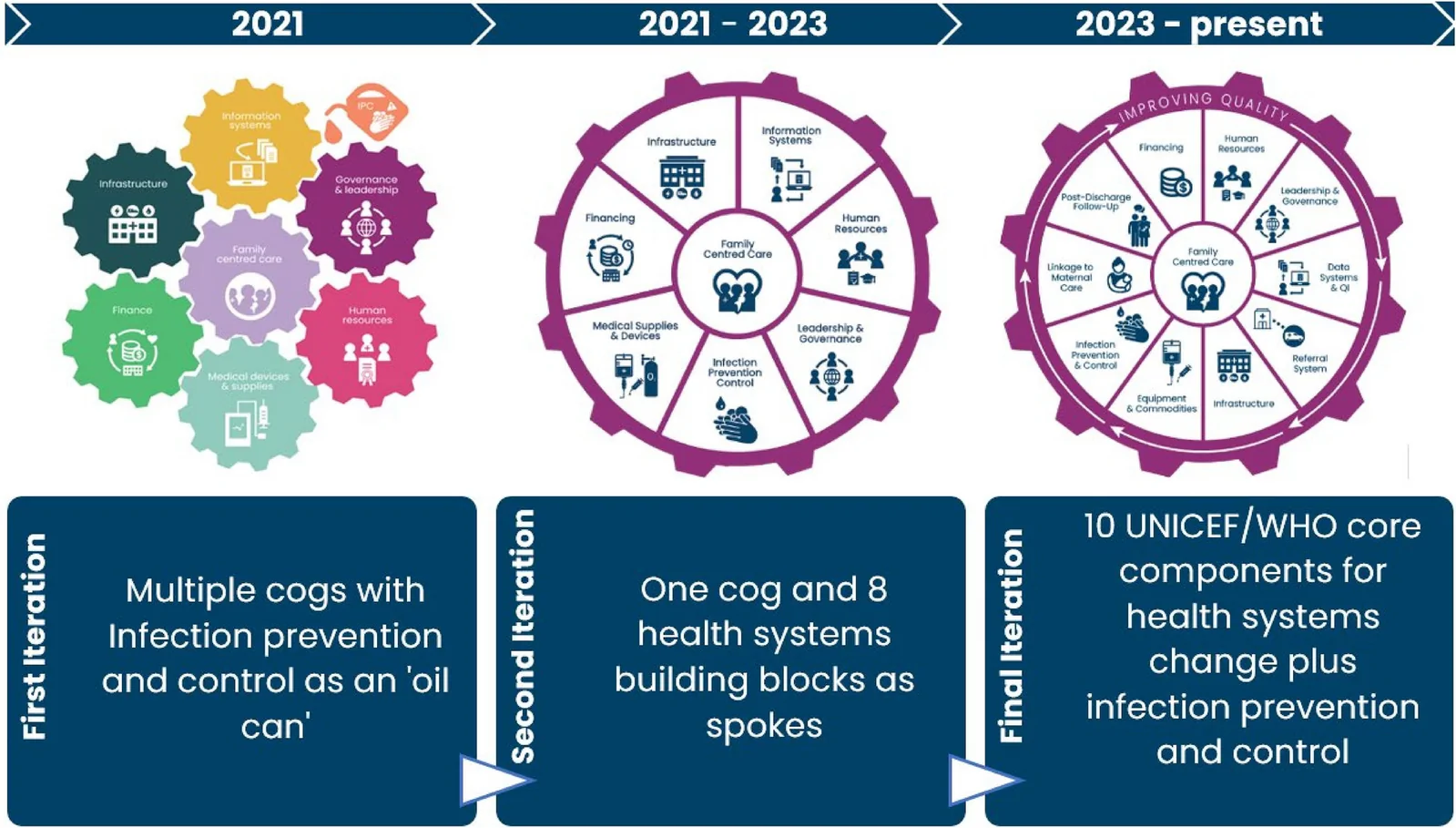
Stages of design of the Newborn Toolkit’s cog incorporating the components for health systems change

Several iterations of the platform visual framework were developed, as shown in Fig. 1. A cog was co-designed as the main shape with an arrow around the components to reflect the dynamism and interrelatedness between the core components necessary to achieve quality care. A unique colour palette was selected for the Newborn Toolkit branding to facilitate visibility and branding independent of NEST360 and UNICEF.

#### Objective 2

As of December 2025, the site hosts a total of 585 tools and 524 readings. Among these resources, 132 tools and 125 readings are available in French. The Toolkit hosts many multilingual tools that have been translated and are available in up to 15 languages.

**Fig. 2:**
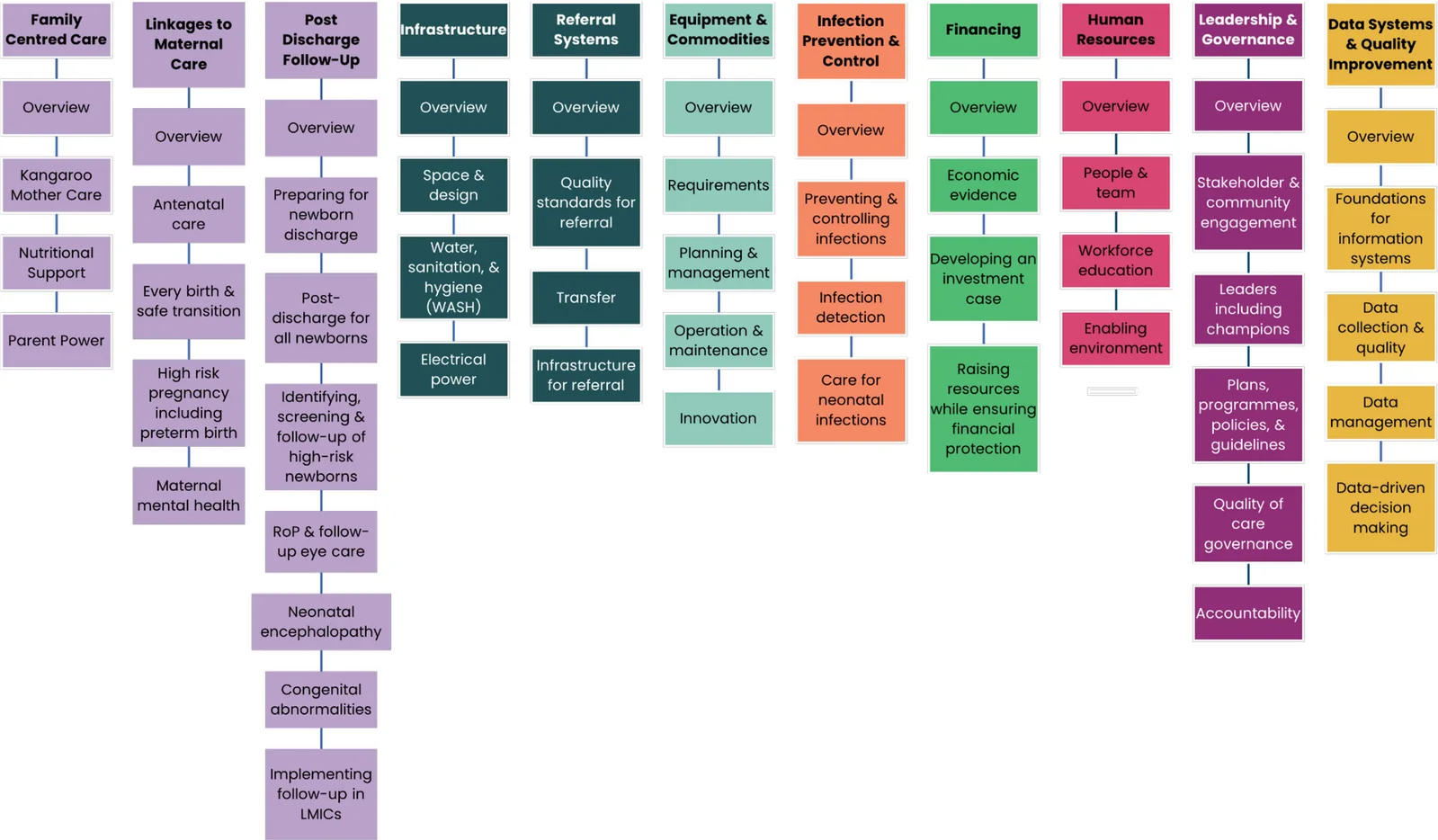
Website technical content organised according to health systems core components plus infection prevention and control

The health systems core component sections contain a narrative overview of the topic and core themes outlining essential steps to improve health delivery in the context of SSNC. For instance, for infection prevention and control, this includes practical strategies to prevent infection, detection and diagnosis, with consideration to facility level and the point of implementation along the continuum of care. Each core component follows the same general layout with an overview section and then 3–8 sub-sections for key themes as outlined in Fig. 2. Within the technical narrative content of each core component, there are inter-linkages to tools, resources, and relevant sections of other core components.

Between 2022–2025, among all health systems core components the most accessed include the following: Data Systems and Quality Improvement, Equipment and Commodities, Infrastructure, Human Resources, and Infection Prevention and Control (Table 1).

**Table 1:** Most accessed resources and tools by WHO and UNICEF core components for health systems strengthening, 2022–2025

Most Accessed	**2022**	**2023**	**2024**	**2025**
1	Human Resources	Infrastructure	Equipment & Commodities	Data Systems & Quality Improvement
2	Data Systems & Quality Improvement	Data Systems & Quality Improvement	Infection Prevention & Control	Infrastructure
3	Leadership & Governance	Equipment & Commodities	Infrastructure	Equipment & Commodities

Most accessed resources between 2022–2025, highlighted in Table 2, included WHO guidelines, neonatal unit floor plans, and NEST360 resources. This includes WHO standards for improving quality of care for small and sick newborns in health facilities, the Tanzania Regional hospital neonatal floor plan, and the ABC Neonatal Devices Planning and Costing Tool developed by NEST360. Country specific tools were also commonly accessed including tools with a contextual framing in India. This includes The India Newborn Action Plan, India complete sample case record forms, and India: Facility Based Newborn Care (FBNC) Facilitator Guide among others. In 2024, NEST360 tools and resources grew in popularity including NEST360 BMC Supplement *— Small and sick newborn care: learning for implementation across Africa and beyond*, NEST360 Health Facility Assessment (HFA) Paper Tools, and NEST360 Facility Quality Improvement Dashboard.

**Table 2:** Most accessed resources on the Newborn Toolkit by year, 2022–2025

Most Accessed	**2022**	**2023**	**2024**	**2025**
1	WHO standards for improving quality of care for small and sick newborns in health facilities (2020)	WHO standards for improving quality of care for small and sick newborns in health facilities (2020)	NHS Toolkit for high-quality neonatal services (2009)	The ICN Code of Ethics for Nurses (2021)
2	WHO infection prevention and control assessment framework at the facility level (2018)	Oxygen Delivery Toolkit: Resources to plan and scale medical oxygen (2023)	The ICN Code of Ethics for Nurses (2021)	NHS Toolkit for high-quality neonatal services (2009)
3	Tanzania Regional hospital neonatal floor plan (2022)	Respiratory Care Equipment Market Report (2020)	Complete sample case record forms (India) (2022)	NNF Clinical Guidelines: Follow-Up for High Risk Newborns (2009)
4	Complete sample case record forms (India) (2022)	Facilitators Guide for Training on Kangaroo Mother Care (2018)	ABC Neonatal Devices Planning and Costing Tool (2024)	Complete sample case record forms (India) (2022)
5	Malawi NICU floor plan (2021)	ABC Neonatal Devices Planning and Costing Tool (2023)	NEST360 BMC Supplement—Small and sick newborn care: learning for implementation across Africa and beyond (2023)	NNF Clinical Practice Guideline: Transport of a Sick Neonate (2023)
6	WHO improving the quality of care for maternal, newborn and child health: implementation guide for national, district and facility levels (2022)	Nigeria Every Newborn Action Plan: A Plan to End Preventable Newborn Deaths in Nigeria (2016)	UNICEF Toolkit for Setting Up Special Care Newborn Units, Stabilisation Units and Newborn Care Corners (2018)	NEST360 Facility Quality Improvement Dashboard (2021)
7	Essential Newborn Care Course Second Edition (2020)	Monitoring the Building Blocks of Health Systems: A Handbook of Indicators and their Measurement Strategies (2010)	Tanzania Regional hospital neonatal floor plan (2022)	The Every Woman Every Newborn Everywhere (EWENE) Dashboard (2023)
8	Implementers’ Voices: Infrastructure (2021)	Close Collaboration with the Parents (2020)	India: Facility Based Newborn Care (FBNC) Facilitator Guide (2014)	UNICEF Toolkit for Setting Up Special Care Newborn Units, Stabilisation Units and Newborn Care Corners (2018)
9	Introducing the WHO quality toolkit: supplemental overview (2022)	Complete sample case record forms (India) (2022)	NEST360 Health Facility Assessment (HFA) Paper Tools (2023)	ABC Neonatal Device Planning and Costing Tool (2024)
10	The India Newborn Action Plan (2014)	Business Models in Respiratory Care (2021)	NEST360 Facility Quality Improvement Dashboard (2021)	WHO standards for improving the quality of care for small and sick newborns in health facilities (2020)

**Fig. 3:**
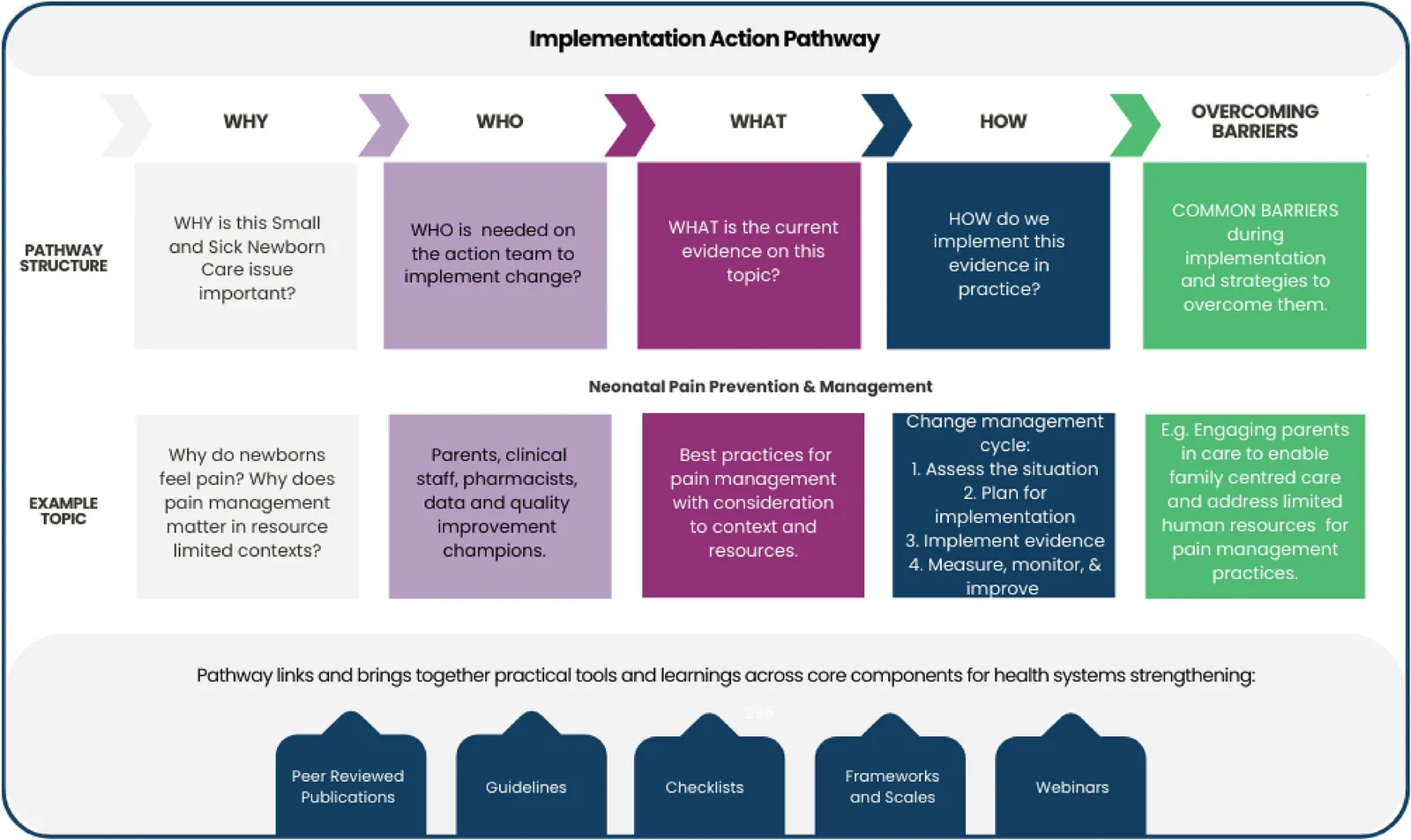
Implementation action pathway structure and neonatal pain prevention and management example

In 2024, to simplify the complexity of systems change and support identification of relevant resources, the Newborn Toolkit developed implementation action pathways. These action pathways include a step-wise process to facilitate implementation of evidence into practice with a specific topic focus, as described in Fig. 3 on the topic of pain management and prevention. Key tools to support implementation are linked along relevant points in the pathway. Pathway themes include Pain Prevention and Management, Quality Kangaroo Mother Care, Neonatal Unit Outbreak Response, and Jaundice Management.

#### Objective 3

The platform called the Implementation Toolkit for SSNC, or Newborn Toolkit, was launched publicly on November 17, 2021 linked to World Prematurity Day.

**Fig. 4:**
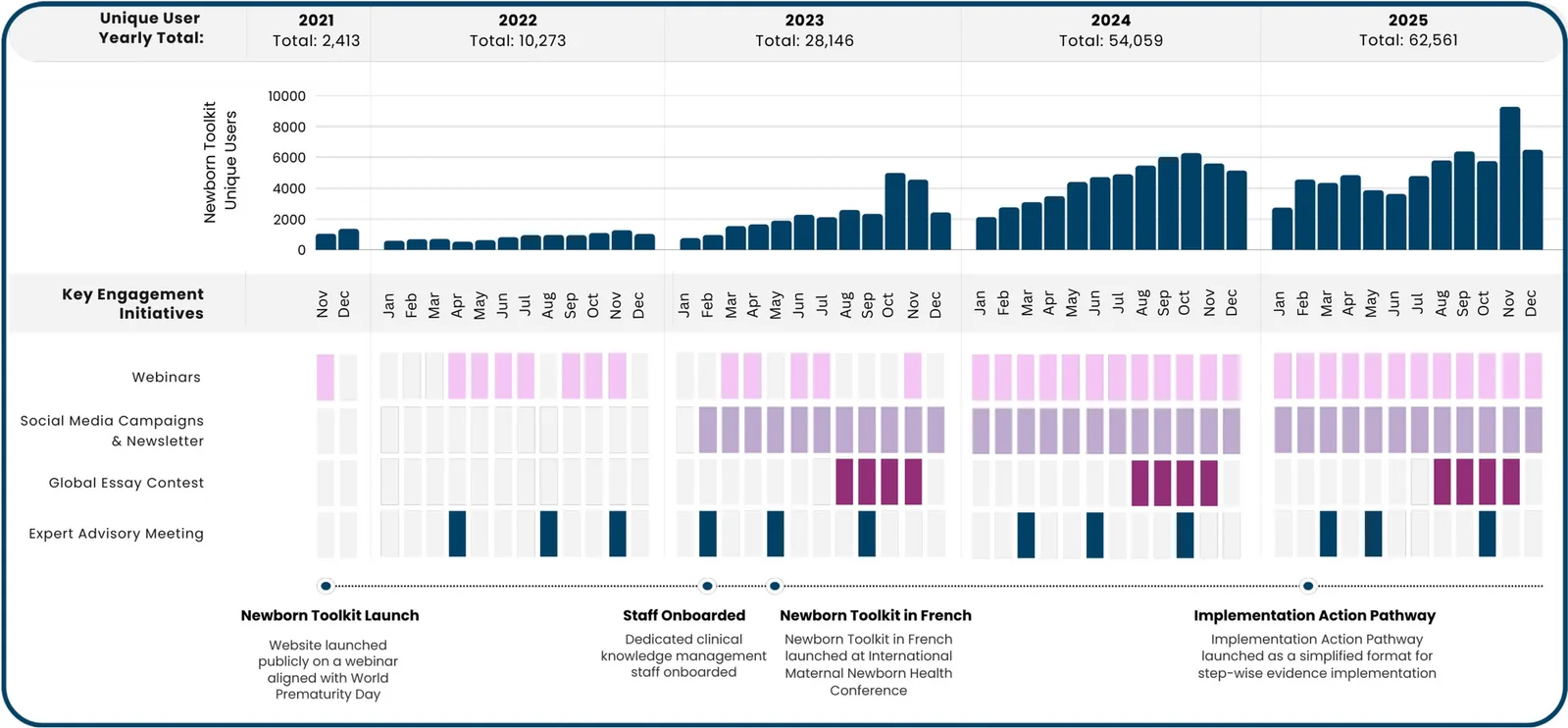
Newborn Toolkit monthly website unique user count and frequency of key engagement initiatives, November 2021 – December 2025

Between November 2021 and December 2025, 157,452 unique users accessed the site (Fig. 4). Unique user growth increased year on year with 10,273 in 2022, 28,146 in 2023, 54,059 in 2024, and 62,561 in 2025 (Fig. 4). October and November in 2023, 2024, and 2025 demonstrated major increases in users with a lower number of users accessing the site in January and February.

**Fig. 5:**
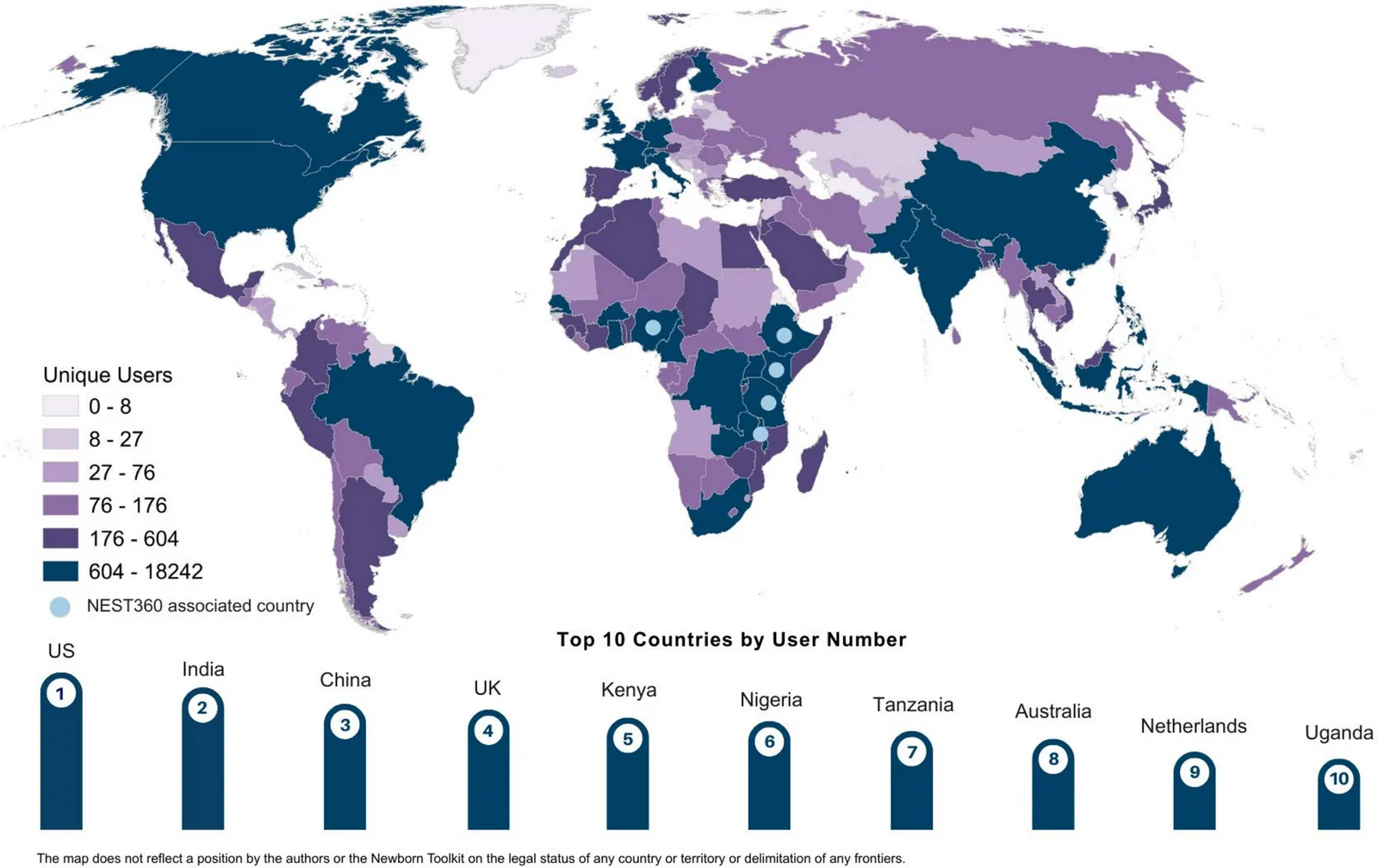
Global distribution of Newborn Toolkit active users, 2022–2025

**Fig. 6:**
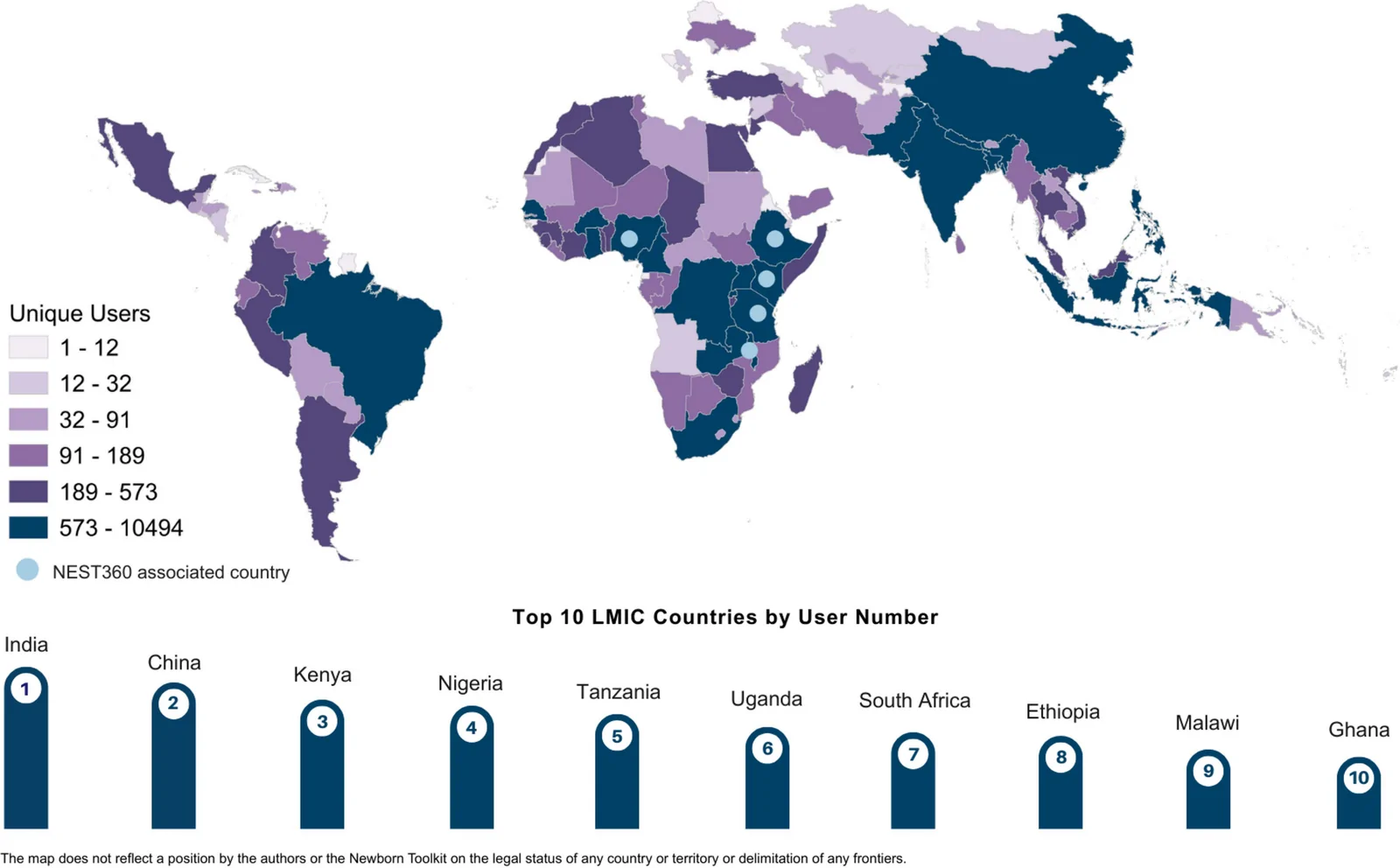
Global distribution of Newborn Toolkit active users among World Bank classified low- and middle-income countries, 2022–2025

As of 2025, users accessed the site from over 198 countries and territories. Figure 5 shows the distribution of users between 2022–2025, among all countries. The following countries were identified as where the Toolkit was most accessed: United States, India, China, United Kingdom, Kenya, Nigeria, Tanzania, Australia, Netherlands and Uganda. Figure 6 displays the World Bank classified LMIC countries where the Toolkit was accessed with top countries including India, China, Kenya, Nigeria, Tanzania, Uganda, South Africa, Ethiopia, Malawi, and Ghana. The top French-speaking countries where the Toolkit was accessed include the following: France, Democratic Republic of the Congo, Rwanda, Cameroon, Senegal, Burkina Faso, Switzerland, Ivory Coast, Benin, and Burundi. Among high-income countries the Toolkit was most accessed from United States, United Kingdom, Australia, Netherlands, Singapore, Ireland, France, Canada, Italy, and Germany.

Since November 17, 2021, 45 webinars have been hosted by the Newborn Toolkit, aligned with the WHO-UNICEF health system core components. Forty-one of these webinars have been delivered in English with live French translation. Three webinars were delivered in French with one additional webinar delivered in Spanish. In 2024 alone, 15 webinars were hosted with attendance reaching nearly 3,500 viewers, through both live sessions and catch-up recordings. In 2025, 12 webinars were hosted with over 2,200 viewers. Among all webinars, the highest in attendance included 1) What’s next for Kangaroo Mother Care before clinical stabilisation, with over 280 live attendees; 2) Kangaroo Mother Care with quality: duration, nutrition, & data that matter, with over 150 live attendees; and 3) Babies and bugs: Innovations to address neonatal sepsis and antimicrobial resistance, with over 120 live attendees. Speakers represented caregiving, clinical, academic, non-governmental, governmental, and private sector backgrounds. This includes WHO, UNICEF, Pan American Health Organization, International Paediatric Association, Council of International Neonatal Nurses, Clinton Health Access Initiative, Global Foundation for the Care of Newborn Infants among others. Speakers featured on the 45 webinars reflected diversity across World Health Organization regions including Africa (*n* = 110), Americas (*n* = 58), European (*n* = 48), South-East Asia (*n *= 22), Western Pacific (*n* = 2), and Eastern Mediterranean (*n* = 1).

## Discussion

This paper describes the co-design, development, and reach of a web platform and linked community of practice for SSNC evidence sharing. The Newborn Toolkit content and community engagement approach is a continuous process informed by regular user feedback and in alignment with data on global newborn health. Continuous inputs have enabled the Newborn Toolkit to adapt and pivot to address current needs of implementers of SSNC in high burden settings. This process has shaped the Newborn Toolkit into an interprofessional educational platform that engages those implementing SSNC globally, with 157,452 unique users from 198 countries and territories between 2021–2025.

### Global distribution of users and practical challenges to uptake

Among low- and middle-income settings, the Toolkit has an active user base in high burden settings including sub-Saharan Africa, South Asia, and South-East Asia [1]. Top countries where users accessed the Newborn Toolkit include the five countries (Kenya, Malawi, Tanzania, Ethiopia, Nigeria) where NEST360, comprising clinicians, biomedical engineers, facility management, and policy makers, is working to improve essential SSNC delivery in over 130 health facilities. The Newborn Toolkit contains many implementation research findings arising from the work undertaken by NEST360 [22, 23]. In the context of managing health conditions, local knowledge production promotes greater sense of ownership, trust, and usable findings that are relevant to context [24]. The linkages between the Toolkit and evidence produced in NEST360-linked health facilities may be an enabler of high engagement among country users. Fostering and leveraging similar partnerships whereby the Newborn Toolkit can host and actively partner to elevate local learning could be an enabler for engaging implementers in other high burden contexts with less engagement. Similarly, reaching and showcasing learning from SSNC implementers, such as nurses or midwives in rural areas is necessary to support greater inclusion among healthcare professionals in more marginalised settings.

Although the target audience of the Newborn Toolkit is for SSNC implementers in low- resource settings, the platform still generates significant interest among high-income countries. Country population size, increased neonatal healthcare professionals, non-governmental organisations, and researchers, may contribute to number of unique users accessing the site by country. Notably, users based in India and the United States were top website users. Both countries have comparatively large populations and health workforces [25, 26]. High-income countries such as the United Kingdom and Australia have bigger neonatal care workforces with lower patient ratios [27, 28].

The Toolkit platform is designed to use low bandwidth and permits users to save offline PDF files; however, low-resource settings experience added challenges with digital accessibility inequities, power shortages, limited internet infrastructure, and lack of information technology support [29] further impeding engagement with the Newborn Toolkit.

### Engagement strategies, particularly regular webinars

With lower platform user engagement initially, significant growth was noted between 2022 to 2025. Growth was enabled by addition of new technical staff including an African neonatal medical doctor and a bilingual English/French neonatal nurse. Technical staff provided more time for the community of practice engagement initiatives from 2023 onwards, notably regular webinars. A strength of the webinars is regular topics based on the 10 core components for SSNC, linking to global action days, such as World Sepsis Day or World KMC Day. Varied speakers also attracted a diverse viewership with different talks tailored by different disciplines. For instance, a webinar sharing interdisciplinary perspectives on devices offered practical guidance for all involved such as health system planners, biomedical engineers and clinicians. Similarly, a webinar on neonatal floor plans or electrical power challenges with an architect and a range of other speakers also resonated across implementers, with relevant cross-disciplinary lessons shared. Targeted engagement also has important implications for evidence uptake. Notably, nurses have reported lack of local contextual evidence as major barriers to evidence implementation [14]. Similarly, a review found that policy makers reported barriers to evidence uptake as a lack of access to good quality relevant research [15].

Fluctuations in website users were generally aligned with Newborn Toolkit engagement activities. Notably, October and November tended to have higher engagement rates; the Newborn Toolkit hosted an annual crowdsourcing essay contest leading up to World Prematurity Day engaging grass-roots implementers of SSNC. More frequent webinars, plus promotion of the Newborn Toolkit at global or national conferences also tended to increase unique user engagement from given countries.

### Engaging a multilingual community

In response to the large representation of French-speaking countries among high burden settings [1], French translation of the Newborn Toolkit content and initiatives was identified as a priority with funding secured in 2023. However, language limitations were noted in the availability of tools and readings. English remains the dominant language of science communication [30, 31]. Compared to over 800 tools available in English, there are only 250 tools in French on the Toolkit. Delays or lack of publication of key evidence in non-English languages create barriers for non-English speakers in accessing and implementing up-to-date evidence [32]. Notably, French and Portuguese speakers based in Africa reported preference for publishing in English as there are more higher impact journals with larger readerships [33]. Similarly, to increase language accessibility, the Newborn Toolkit hosted webinars featuring French only speakers; however, these webinars were poorly attended by both French and English speakers. Webinars featuring English speakers or combined English/French speakers were better attended by French-speakers compared to webinars featuring only French-speakers. This preference for English-speaking webinars could be attributed to the webinar topics with topic selection often guided by latest evidence related to SSNC. This evidence is often published first in English and thus presented and discussed by English-speakers on webinars.

The Toolkit narrative overview content and engagement initiatives are currently only offered in English and French. Within Africa alone, there are over 1,000 languages spoken with many regional dialects [33]. Fewer languages and limited tailoring to regional cultural norms may limit the uptake of the Toolkit particularly among those speaking minority languages. Portuguese and Spanish, followed by Swahili, Arabic, and Hindi, have been selected as priority languages for translation of the Toolkit reflecting languages spoken in regions with high newborn mortality. Translation is a priority focus for further funding applications; however, funding opportunities in non-English languages for newborn health are fewer [34]. Advancements in AI-enabled translation permit faster translation and accessibility, however limitations in the accuracy of medical evidence translation, may pose ethical and safety challenges [35, 36], particularly in the context of vulnerable newborns. To bridge this language divide, journals and organisations that produce health guidance could consider translation of evidence to non-English languages, with post-translation review, particularly for findings that have potential for high impact in non-English speaking settings.

### Simplifying complexity

Organisation and delivery of content have important implications for uptake and use of evidence in practice. Enablers of engagement linked to website design include ease of navigation, presence of graphics, website organisation, content utility, purpose, simplicity, and readability [37]. In the context of health guidance, a review identified that challenges in evidence uptake were linked to lack of guideline availability, difficultly in locating guidance, lack of staff training on guidance updates, and multiple different international and national guidelines [38]. Establishing and improving quality of care delivery across multiple components of a health system is a complex process that requires nuance stemming from a large body of evidence [39]. A strength of the Newborn Toolkit is its robust repository of evidence for quality care delivery, located in one convenient place. However, with over 1,100 resources, for users, it can be overwhelming to discern which tools and evidence are best. Implementation Action Pathways provided a means to support uptake of evidence in a simplified step-wise approach. These pathways also identified varied target users and integrated implementation considerations for different health professionals into the approach for applying evidence in practice.

### Sustainability

The long-term sustainability of the Newborn Toolkit was co-designed from inception to bring in wide ownership and a range of funders. The platform has a deliberately neutral domain name (as opposed to NEST360, UNICEF, or LSHTM). Content is clearly linked to United Nations (UN) guidelines and WHO/UNICEF structural framework as an intentional choice, enabling a broad coalition of governments, implementing partners and donors to contribute without competing over institutional ownership. The Newborn Toolkit is co-designed by UNICEF and NEST360 as an open-access, online platform enabling implementers globally. This co-ownership with UNICEF was intended as a direct pathway to sustainability. This model is analogous to DHIS2, which is governed by a coalition of global health partners including UNICEF, Norad, PEPFAR, and the Global Fund [40].

The Newborn Toolkit has an Expert Advisory Team consisting of a wide network of partners; WHO, UNICEF, PMNCH, plus the leading healthcare professional organisations and many others, some of whom are funders, and this enables wide ownership to promote sustainability. The Toolkit was designed as a Digital Public Good and we plan to formalise this through registry under the UN Secretary-General's Roadmap for Digital Cooperation [41], providing both legitimacy and access to multi-stakeholder SDG-aligned funding.

Continued development of the platform as free open-source, irrespective of who hosts it is dependent on funding since there are modest costs for domain hosting and licences, but growth of content and community requires responsiveness with staff time. The platform domain, financial management, and core staff are hosted at the London School of Hygiene & Tropical Medicine. Funding so far has been from a portfolio of private donors and competitive government funders. Despite major worldwide usage and interest in the platform, a key challenge is the reliance on donor investment, in an environment where global health funding has contracted.

### Strengths and limitations

A strength of the Newborn Toolkit includes the three-step systematic co-development process including continuous feedback incorporated to target gaps and refine website content. Development of the website content was guided by a framework, WHO/UNICEF’s health systems ten core components, and was informed by input from over 300 implementers of SSNC honing a breadth and depth of topic experience. The Toolkit hosts a large repository of practical tools in over 15 languages.

A limitation includes the inability to measure or attribute direct impact of the Toolkit on health outcomes. Attribution of causality would not be possible. Evaluation of changes in user knowledge would be possible, as would qualitative data on perceived value. Beyond engagement with the Toolkit, and downloading tools or attending webinars, local level change in implementation also requires meaningful health systems improvement and investment of time and resources. User data is dependent on Google analytics which has strengths and weaknesses. Data from 2021 was not included in site analytics as the website was only launched publicly in November 2021. Lower unique users in 2022 was likely to be real with less engagement noting that tools and content was being expanded and there were fewer webinar offerings during this time.

## Conclusions

With 2.3 million neonatal deaths estimated annually, and 65 countries off-track to reaching the SDG for newborn survival by 2030, progress on SSNC needs to be accelerated. By providing curated and packaged relevant evidence combined with sharing diverse local learning (much of which has not been published), the Newborn Toolkit aims to enable data-driven decision making. Higher coverage and quality of implementation of known evidence is a pre-requisite for faster progress in high-burden settings. This requires more targeted language offerings of evidence, and more opportunities for active cross-country learning to truly close the know-do gap.

## Data Availability

The dataset is available in the public domain on the Implementation Toolkit for Small and Sick Newborn Care (https://newborntoolkit.org/). For the purpose of open access, the author has applied a ‘Creative Commons Attribution’ (CC BY) license to any Author Accepted Manuscript version arising.

## Abbreviations

LMIC: Low- and Middle- Income Country
LSHTM: London School of Hygiene & Tropical Medicine
NEST360: Newborn Essential Solutions and Technologies 360
ROP: Retinopathy of Prematurity
SEO: Search Engine Optimisation
SSNC: Small and Sick Newborn Care
UN: United Nations
UNICEF: United Nations Children’s Fund
WHO: World Health Organization
WPML: Word Press Multilingual

## References

[1] UNIGME. Levels and Trends in Child Mortality. 2025. https://data.unicef.org/resources/levels-and-trends-in-child-mortality-2024/. Accessed 25 May 2025.

[2] March of Dimes, PMNCH, Save the Children, WHO. The Global Action Report on Preterm Births. 2012. https://iris.who.int/bitstream/handle/10665/44864/9789241503433_eng.pdf?sequence=1. Accessed 25 May 2025.

[3] WHO. Newborn mortality. 2024. https://www.who.int/news-room/fact-sheets/detail/newborn-mortality#:~:text=It%20is%20possible%20to%20improve,of%20small%20and%20sick%20newborns. Accessed 25 May 2025.

[4] WHO. Essential Newborn Care. 2025. https://www.who.int/teams/maternal-newborn-child-adolescent-health-and-ageing/newborn-health/essential-newborn-care#:~:text=Essential%20newborn%20care%20includes%3A,Resuscitation%20when%20needed.

[5] WHO. Survive and Thrive: Transforming care for every small and sick newborn. 2019. https://www.who.int/publications-detail-redirect/survive-and-thrive-transforming-care-for-every-small-and-sick-newborn. Accessed 25 May 2025.

[6] WHO-UNICEF Expert and Country Consultation on Small and/or Sick Newborn Care Group. A comprehensive model for scaling up care for small and/or sick newborns at district level–based on country experiences presented at a WHO-UNICEF expert consultation. J Glob Health. 2023;21(13):03023. 10.7189/jogh.13.03023. 10.7189/jogh.13.03023 PMC10120390 37083313

[7] Sacks E, Morrow M, Story WT, Shelley KD, Shanklin D, Rahimtoola M, et al. Beyond the building blocks: integrating community roles into health systems frameworks to achieve health for all. BMJ Glob Health. 2019;3:e001384. 10.1136/bmjgh-2018-001384. 31297243 10.1136/bmjgh-2018-001384 PMC6591791

[8] Van Olmen J, Marchal B, Damme WV, Kegels G, Hill PS. Health systems frameworks in their political context: framing divergent agendas. BMC Public Health. 2012;12:774. 10.1186/1471-2458-12-774. 22971107 10.1186/1471-2458-12-774 PMC3549286

[9] WHO. Systems Thinking for Health Systems Strengthening. 2009. https://evidence-impact.org/storage/165/Systems-Thinking-for-Health-Systems-Strengthening.pdf. Accessed 25 May 2025.

[10] Kruk ME, Gage AD, Arsenault C, Jordan K, Leslie HH, Roder-Dewan S, et al. High-quality health systems in the Sustainable Development Goals era: time for a revolution. Lancet Glob Health. 2018;6(11):E1196–252. 10.1016/S2214-109X(18)30386-3. 30196093 10.1016/S2214-109X(18)30386-3 PMC7734391

[11] Murless-Collins S, Ezeaka VC, Masoud NS, Walker K, Rhoda NR, Keenan W, et al. Born too soon: care for small and sick newborns, evidence for investment and implementation. Reprod Health. 2025;22:114. 10.1186/s12978-025-02032-y. 40555990 10.1186/s12978-025-02032-y PMC12188657

[12] Greenway K, Butt G, Walthall H. What is a theory-practice gap? An exploration of the concept. J Nurs Educ Pract. 2019;34:1–6. 10.1016/j.nepr.2018.10.005. 10.1016/j.nepr.2018.10.005 30393025

[13] Donohue JF, Elborn JS, Lansberg P, Javed A, Tesfaye S, Rugo H, et al. Bridging the “know-do” gaps in five noncommunicable diseases using a common framework driven by implementation science. J Healthc Leadersh. 2023;15:103–19. 10.2147/JHL.S394088. 37416849 10.2147/JHL.S394088 PMC10320809

[14] Pitsillidou M, Roupa Z, Farmakas A, Noula M. Factors affecting the application and implementation of evidence-based practice in nursing. Acta Inform Med. 2021;29:281–7. 10.5455/aim.2021.29.281-287. 35197664 10.5455/aim.2021.29.281-287 PMC8800576

[15] Oliver K, Innvar S, Lorenc T, Woodman J, Thomas J. A systematic review of barriers to and facilitators of the use of evidence by policymakers. BMC Health Serv Res. 2014;14:2. 10.1186/1472-6963-14-2. 24383766 10.1186/1472-6963-14-2 PMC3909454

[16] Kothari A, Hovanec N, Hastie R, Sibbald S. Lessons from the business sector for successful knowledge management in health care: a systematic review. BMC Health Serv Res. 2011;11:173. 10.1186/1472-6963-11-173. 21787403 10.1186/1472-6963-11-173 PMC3157420

[17] Yazdani S, Bayazidi S, Mafi AA. The current understanding of knowledge management concepts: a critical review. Med J Islam Repub Iran. 2020;34:127. 10.34171/mjiri.34.127. 33437723 10.34171/mjiri.34.127 PMC7787024

[18] El-Jardali F, Bou-Karroum L, Hilal N, Hammoud M, Hemadi N, Assal M, et al. Knowledge management tools and mechanisms for evidence-informed decision-making in the WHO European Region: a scoping review. Health Res Policy and Syst. 2023;21:113. 10.1186/s12961-023-01058-7. 37907919 10.1186/s12961-023-01058-7 PMC10619313

[19] UNICEF, UNDP, World Bank, WHO. TDR Implementation Research Toolkit (Second Edition). 2025. https://adphealth.org/irtoolkit/. Accessed 25 May 2025.

[20] Google. Understanding user metrics. https://support.google.com/analytics/answer/12253918?hl=en. 2026. Accessed 21 May 2026.

[21] Google. Known bot-traffic exclusion. https://support.google.com/analytics/answer/9888366?hl=en. 2026. Accessed 21 May 2026.

[22] Murless-Collins C, Kawaza K, Salim N, Molyneux EM, Chiume M, Aluvaala J, et al. Blood culture versus antibiotic use for neonatal inpatients in 61 hospitals implementing with the NEST360 Alliance in Kenya, Malawi, Nigeria, and Tanzania: a cross-sectional study. BMC Pediatr. 2023;23:568. 10.1186/s12887-023-04343-0. 37968606 10.1186/s12887-023-04343-0 PMC10652421

[23] Tarus A, Msemo G, Kamuyu R, Shamba D, Kirby RP, Palamountain KM, et al. Devices and furniture for small and sick newborn care: systematic development of a planning and costing tool. BMC Pediatr. 2023;23:566. 10.1186/s12887-023-04363-w. 37968613 10.1186/s12887-023-04363-w PMC10652422

[24] Grindell C, Coates E, Croot L, O’Cathain A. The use of co-production, co-design and co-creation to mobilise knowledge in the management of health conditions: a systematic review. Health Serv Res. 2022;22:877. 10.1186/s12913-022-08079-y. 10.1186/s12913-022-08079-y PMC9264579 35799251

[25] Karan A, Negandhi H, Hussain S, Zapata T, Mairembam D, De Graeve H, et al. Size, composition and distribution of health workforce in India: why, and where to invest? Hum Resour Health. 2021;19:39. 10.1186/s12960-021-00575-2. 33752675 10.1186/s12960-021-00575-2 PMC7983088

[26] Horowitz E, Hudak ML, Peña M, Vinci RJ, Savich R. Child Health Needs and the Neonatal-Perinatal Medicine Workforce: 2020–2040. Pediatr. 2024;153:e2023063678O. 10.1542/peds.2023-063678O. 10.1542/peds.2023-063678O 38300002

[27] McHugh MD, Aiken LH, Sloane DM, Windsor C, Douglas C, Yates P. Effects of nurse-to-patient ratio legislation on nurse staffing and patient mortality, readmissions, and length of stay: a prospective study in a panel of hospitals. The Lancet. 2021;397(10288):1905–13. 10.1016/S0140-6736(21)00768-6. 10.1016/S0140-6736(21)00768-6 PMC8408834 33989553

[28] Australian Government Department of Health. Medical Workforce. 2016. https://hwd.health.gov.au/webapi/customer/documents/factsheets/2016/Medical%20workforce%20factsheet%202016.pdf. Accessed 25 May 2025.

[29] Barteit S, Albrecht J, Banda SS, Bärnighausen T, Bowa A, Chileshe G, et al. E-learning for medical education in Sub-Saharan Africa and low-resource settings: viewpoint. JMIR. 2019;21(1):e12449. 10.2196/12449. 30626565 10.2196/12449 PMC6329426

[30] Drubin DG, Kellogg DR. English as the universal language of science: opportunities and challenges. Mol Biol Cell. 2012;23(8):1399. 10.1091/mbc.E12-02-0108. 22499829 10.1091/mbc.E12-02-0108 PMC3341706

[31] Rasmussen LN, Montgomery P. The prevalence of and factors associated with inclusion of non-English language studies in Campbell systematic reviews: a survey and meta-epidemiological study. Syst Rev. 2018;7:129. 10.1186/s13643-018-0786-6. 30139391 10.1186/s13643-018-0786-6 PMC6107944

[32] Hommes F, Monzó HB, Ferrand RA, Harris M, Hirsch LA, Besson EK, et al. The words we choose matter: recognising the importance of language in decolonising global health. Lancet Glob Health. 2021;9(7):e897–8. 34143986 10.1016/S2214-109X(21)00197-2

[33] Yarmoshuk AN, Mloka D, Touré SF, Sharma V, Wanji S. Research into language-based equity in African health science research. The Wellcome Trust; 2021. https://cms.wellcome.org/sites/default/files/2021-06/language-based-equity-in-african-health-science-research.pdf. Accessed 25 May 2025.

[34] Allison LE, Ruysen H, Sánchez Alva MJ, Agravat P, Loucaides E, Kumar MB, et al. Who gets funded? Global analysis of research funding for newborn health and stillbirth in fragile and non-English speaking countries, 2016–2020. SSM Health Syst. 2025;5:100144. 10.1016/j.ssmhs.2025.100144.

[35] Genovese A, Borna S, Gomez-Cabello CA, Haider SA, Prabha S, Forte AJ, et al. Artificial intelligence in clinical settings: a systematic review of its role in language translation and interpretation. Ann Transl Med. 2024;12:117. 10.21037/atm-24-162. 39817236 10.21037/atm-24-162 PMC11729812

[36] Lion KC, Lin Y, Kim T. Artificial Intelligence for Language Translation The Equity Is in the Details. JAMA. 2024;332(17):1427–8. 10.1001/jama.2024.15296. 39264601 10.1001/jama.2024.15296

[37] Garett R, Chiu J, Zhang LY, Young SD. A literature review: website design and user engagement. J Commun Media Technol. 2016;6(3):1–14. PMC4974011 27499833

[38] RaghunathanI K, East C, Poudel K. Barriers and enablers for implementation of clinical practice guidelines in maternity and neonatal settings: a rapid review. PLoS ONE. 2024;19(12):e0315588. 10.1371/journal.pone.0315588. 39680550 10.1371/journal.pone.0315588 PMC11649122

[39] Mianda S, Todowede O, Schneider H. Service delivery interventions to improve maternal and newborn health in lowand middle-income countries: scoping review of quality improvement, implementation research and health system strengthening approaches. BMC Health Serv Res. 2023;23:1223. 10.1186/s12913-023-10202-6. 37940974 10.1186/s12913-023-10202-6 PMC10634015

[40] DHIS2. Sustainability. https://dhis2.org/about-2/ . 2026. Accessed 21 May 2026.

[41] United Nations. Road map for digital cooperation: implementation of the recommendations of the High-level Panel on Digital Cooperation. 2020. https://www.un.org/en/content/digital-cooperation-roadmap/. Accessed 21 May 2026.

